# Methotrexate therapy of T-cell large granular lymphocytic leukemia impact of *STAT3* mutation

**DOI:** 10.18632/oncotarget.11360

**Published:** 2016-08-17

**Authors:** Zhi-Yuan Qiu, Lei Fan, Rong Wang, Robert Peter Gale, Hua-Jin Liang, Man Wang, Li Wang, Yu-Jie Wu, Chun Qiao, Yao-Yu Chen, Wei Xu, Jun Qian, Jian-Yong Li

**Affiliations:** ^1^ Department of Hematology, The First Affiliated Hospital of Nanjing Medical University, Jiangsu Province Hospital, Collaborative Innovation Center for Cancer Personalized Medicine, Nanjing, China; ^2^ Department of Oncology, The Affiliated People's Hospital of Jiangsu University, Zhenjiang, Jiangsu, China; ^3^ Haematology Research Centre, Division of Experimental Medicine, Department of Medicine, Imperial College London, London, United Kingdom; ^4^ Department of Hematology, The Affiliated People's Hospital of Jiangsu University, Zhenjiang, Jiangsu, China

**Keywords:** T-cell large granular lymphocytic leukemia, methotrexate, STAT3

## Abstract

T-cell large granular lymphocytic leukemia (T-LGLL) is a rare haematologic neoplasm. Consequntly, there are no large prospective studies of therapy and no uniform therapy recommendations. We analyzed data from 36 subjects receiving methotrexate alone (*N* = 27) or with prednisone (*N* = 9) as initial therapy. 31 subjects responded (86%, 95% confidence interval [CI], 73, 95%) with 8 complete responses and 23 partial responses. Median time-to-response was 3 months (range, 1–5 months). Median response duration was 20 months (range, 2–55 months). β_2_-microoglobulin (β_2_-MG) and erythrocyte sedimentation rate (ESR) decreased significantly post-therapy (P < 0.0001). Pure red cell aplasia (PRCA) was present in 18 subjects (50%) of our subjects and responded well to methotrexate. 26 subjects (72%) were tested for *STAT3* mutation. 9 with a mutation had a median treatment-free survival of 5 months (range, 0.5–13 months), significantly briefer than that of 17 subjects without a *STAT3* mutation (19 months, range, 3–97 months; *P* = 0.012; log-rank test). Methotrexate with or without prednisone is an effective initial therapy of persons with T-LGLL with wild-type *STAT3*.

## INTRODUCTION

T-cell large granular lymphocytic leukemia (T-LGLL) is a lympho-proliferative neoplasm of cytotoxic T cells [1]. There is no standard therapy but immune suppression is commonly used when therapy is needed. The three most commonly used drugs are methotrexate, cyclosporine and cyclophosphamide. Treatment outcomes are heterogeneous and there is only one comparative study which shows immune suppression is effective and mutational profiling predicts response [2].

T-LGLL can present as failure of hematopoiesis or immune-mediated destruction of one or several cell lines including RBCs, neutrophils, and platelets. T-LGLL associated pure red cell aplasia (PRCA) accounts for a significant portion of secondary PRCA [3]. Because T-LGLL and T-LGLL associated PRCA are rare optimal long-term outcomes after immune suppressive therapy are controversial. We report results using methotrexate as initial therapy of T-LGLL in 36 subjects most of whom also had PRCA. Correlative studies were conducted to determine if biomarkers or genetic analysis could predict therapy response.

## RESULTS

### Clinical variables

Data on the 36 subjects are displayed in Table 1. There were 20 males. Median age at diagnosis was 60 years (range, 38–89 years). Median follow-up is 39 months (range, 8–94 months).

**Table 1:** Subject-related variables

N	36
Male	20
Age (median;range)	60 (38–89)
Symptoms at diagnosis	35
PRCA	18
Neutrophils < 1.5 ×10E+9/L	24
Neutrophils < 0.5×10E+9/L	1
Hemoglobin < 110 g/L	34
Platelet < 100 × 10E+9/L	7
LGL × 10E+9/L Mean ± SD	2.9 ± 1.9
Hepatomegaly	3
Splenomegaly	18
Lymphadenopathy	2
LDH >250U/L	18
RF	3
ANA	7
ESR > 20 mm/h	24
β2-MG > 3.0 mg/L	23

RF, rheumatoid factor; PRCA, pure red cell aplasia; LGL, large granular lymphocyte; ANA, anti-nuclear antibody; β2-MG, β2-microglobulin; ESR, erythrocyte sedimentation rate.

### Laboratory results

Four subjects had anaemia (Hemoglobin < 110 g/L) and decreases in neutrophils (< 1.5 × 10E + 9/L) and platelets (< 100 × 10E + 9/L). 24 had only neutropenia, severe in 1. Thrombocytopenia was present in 7 subjects. Anaemia was present in 34 (Table 1). 18 subjects had PRCA (Table 1). 23 subjects tested had normal cytogenetics. Three subjects had a positive rheumatoid factor test one of whom had rheumatoid arthritis. All subjects had clonal rearrangement of the T-cell receptor.

### Immune phenotypes and Vβ expression

**Figure 1:**
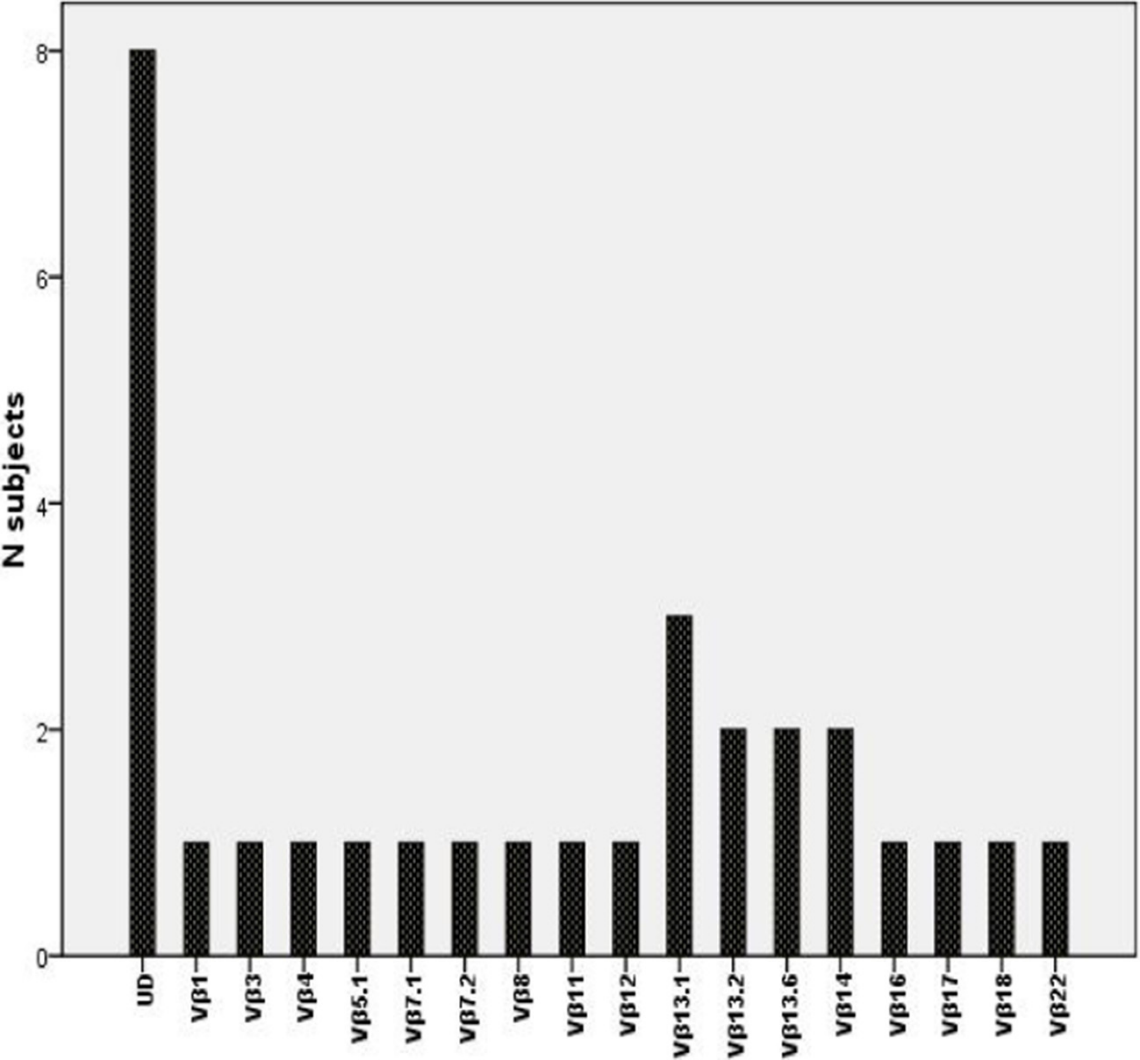
TCR-vβ (UD: un-dectable)

34 subjects had the predominant LGL phenotype (CD3+CD8+CD57+CD56−) and 2 subjects with CD3+CD4+CD57+CD56−. 33 subjects were TCRαβ+ and 3 subjects were TCRγδ+. Flow cytometric variable β-chain repertoire (FC-Vβ) analysis was done in 30 subjects. 22 (73%) subjects had a restricted Vβ reactivity pattern; Vβ 13.1 was relatively frequent (Figure 1). There was complete lack of Vβ expression in 8 subjects.

### Response

27 subjects received methotrexate only and 9, methotrexate and prednisone. 31 subjects (86% [73, 95%]) responded with 8 complete responses and 23 partial responses. We compared initial characteristics of non-responders, partial responders and complete responders, but no differences were found in terms of age, gender, splenomegaly, hepatomegaly, symptoms at diagnosis, LDH, LGL count, neutropenia, anemia and thrombocytopenia. Median teatment-free-survival was 13.1 months (range, 0.5–97 months). Median time-to-response (TTR) was 3 months (range, 1–5 months). Median response duration was 20 months (range, 2–55 months). 9 subjects receiving methotrexate and prednisone had a median time-to-response (TTR) of 2.3 months (range, 1–4 months), briefer than the TTR in the 26 subjects receiving methotrexate alone, 2.7 months (range, 1–5 months; *P* = 0.27). 3 subjects who maintained their response were lost to follow-up. 30 subjects maintained their response. One subject died from disease progression. By the end of follow-up, 22 subjects were receiving methotrexate (16 subjects received continuous methotrexate and 6 received lower doses methotrexate). 4 subjects maintained their response after discontinuing methotrexate. 4 subjects relapsed and received cyclosporine of whom 3 responded (partial responses). The non-responder to cyclosporine then received low-dose oral cyclophosphamide achieving a complete response.

In the 18 subjects with PRCA there were 6 complete and 11 partial responses with an overall response rate of 94% (95% confidence interval, 73–100%). Median time-to-response was 3 months (range, 1–5 months). Median response duration was 20 months (range, 5–55 months). Response rate in subjects with PRCA was not significantly different than in all subjects.

### Serum biomarkers

We also assessed dynamic changes of ESR and β_2_-MG in serum of subjects as a marker of therapy response. Baseline levels (mean ± SEM) of serum β_2_-MG and ESR levels were 4.67 ± 2.76 mg/L and 61.2 ± 41.1 mm/h. Post-therapy values at the time of best response were 2.76 ± 1.53 mg/L (*P* = 0.003) and 20.1 ± 21.0 mm/h (*P* < 0.001).

### Toxicity

Adverse events were uncommon and none required stopping therapy. There were 2 grade-1 toxicities, one an alanine aminotransferase increase and one a total bilirubin increase each in 1 subject. Grade-2 toxicities included oral mucositis (1 subject) and nausea (3 subjects).

### *STAT3* mutation

**Figure 2:**
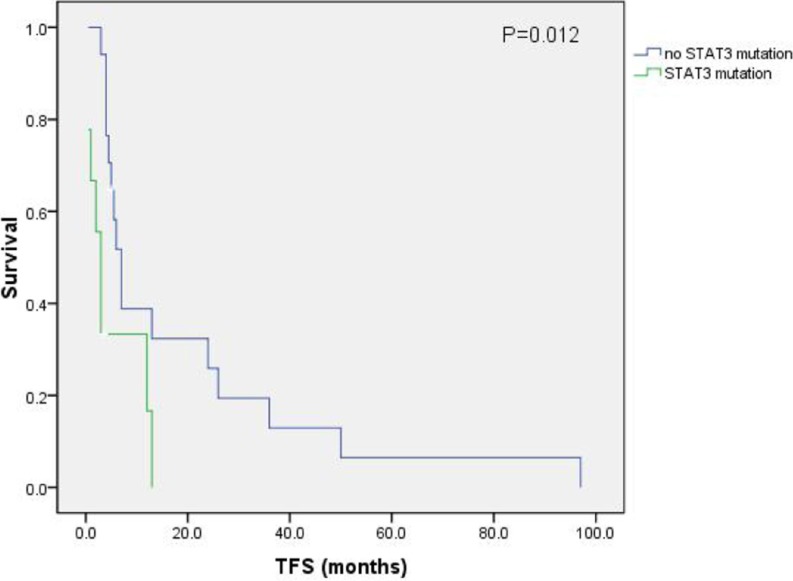
Treatment-free-survival

26 subjects were tested for *STAT3* mutation. 9 had mutations including *STAT3*^Y640F^ (*N* = 4), *STAT3*^D661Y^ (*N* = 2), *STAT3*^V671F^ (*N* = 1), *STAT3S*^614R^ (*N* = 1) and *STAT3*^E616V^ (*N* = 1). Subjects with *STAT3* mutation had briefer treatment-free survival than subjects with wild-type STAT3 (5 mo [range, 0.5–13 mo] *vs*. 19 mo [range, 3–97 mo]; *P* = 0.012; Figure 2). Time-to-response and response-duration were also briefer in subjects with a *STAT3* mutation but these differences were not significant. There were too few subjects to test this association in multivariate analyses. Comparison of the clinical variable in subjects with or without STAT3 mutation is shown in Table 2.

**Table 2:** Clinical differences between patients with or without *STAT3* mutation

Variable	Patients with STAT3 mutation (%)	Patients without STAT3 mutation (%)	*P*
Gender			1.0
Male	4 (44.4)	9 (47.1)	
Female	5 (55.6)	8 (52.9)	
Age (years)			0.53
Mean ± SD	58.1 ± 9.5	55.7 ± 10.4	
LDH >250U/L	6 (66.7)	7(41.2)	0.41
β2-MG >3.0 mg/L (n=24)	9 (100.0)	10(66.7)	0.12
Symptoms at diagnosis, no. (%)	9 (94.1)	16 (54.5)	1.0
Neutropenia, no. (%)	7 (77.8)	12 (70.6)	1.0
Anemia, no. (%)	8 (88.9)	17 (100)	0.35
Thrombocytopenia, no. (%)	1 (11.1)	1 (5.9)	1.0
LGL count in PB, ×10E+9/L			
Mean ± SD	3.89 ± 2.44	2.83 ± 1.72	0.095
Hepatomegaly, no. (%)	2 (22.2)	0 (0)	0.11
Splenomegaly, no. (%)	7 (77.8)	7 (41.2)	0.11
PRCA, no. (%)	7 (77.8)	8 (47.1)	0.21

PB: peripheral blood; *STAT3*: signal transducer and activator of transcription 3.

## DISCUSSION

We report a high response rate with methotrexate with or without prednisone as initial therapy of T-LGLL. Subjects with associated PRCA had correspondingly high response rates and long response durations. Others report similar response rate but many more relapses [4–6]. Time-to-response of subjects receiving methotrexate and prednisone was briefer subjects receiving methotrexate only but this allocation was not randomized and requires confirmation. No responder relapsed immediately after discontinuing prednisone. Response rates were similar in persons with and without PRCA.

30–40% of persons with T-LGLL have mutations in *STAT*3 [7, 8]. We found *STAT3* mutations in 9 of 26 subjects (35%). Like some, but in contrast to other reports, we found an association between *STAT3* mutation and PRCA [8, 9]. We also found differences in time-to-response and treatment-free survival in subjects with and without *STAT3* mutations. Others reported *STAT3*^Y640F^ is associated with response to methotrexate [2]. These data suggest screening for *STAT3* mutations may be useful.

There are important limitations to our study. Numbers of subjects is small compromising our power to detect differences. Although the series was consecutive we cannot exclude referral biases. Assignment to prednisone was not random. Finally, there were too few subjects to test the association between *STAT3* mutation and treatment-free survival. Because of these limitations our conclusions require validation. In summary, methotrexate alone or combined with prednisone is an effective initial therapy of T-LGLL with high response rates and durable responses. Persons with associated *STAT3* mutation had worse outcome than those with wild-type *STAT3*.

## MATERIALS AND METHODS

### Subjects

36 consecutive subjects with newly-diagnosed, untreated T-LGLL were seen February, 2005 to October, 2014 in the First Affiliated Hospital of Nanjing Medical University (Jiangsu Province Hospital). Subjects gave written informed consent and the University and Institutional Review Boards approved the study which conformed to the Declaration of Helsinki. Diagnosis of T-LGLL was based on WHO criteria including: (1) blood morphology; (2) presence of an abnormal cytotoxic lymphocyte population with expression of CD3, CD8 and CD57; (3) LGL concentration > 2 × 10E + 9/L; (4) T-cell clonality; and (5) persistence > 6 months. PRCA was defined as reported [10].

### Laboratory data

Data collected at diagnosis included age, sex, symptoms, hepatomegaly, splenomegaly, lymphadenopathy, WBC, hemoglobin concentration, neutrophil, platelet, lymphocyte and LGL levels, lactate dehydrogenase (LDH), erythrocyte sedimentation rate (ESR) and serum β_2_-microglobulin (β_2_-MG).

### Flow cytometric analysis for immune phenotype and T-cell receptor Vβ repertoire (TCR-Vβ)

Immune phenotypic analysis of PB and/or bone marrow (BM) samples were determined using specific antibodies against CD2 (Leu-5b), CD3 (SK7), CD4 (Leu-3a), CD5 (Leu-1), CD7 (3A1), CD8 (Leu-2a), CD16 (3G8), CD56 (NCAM16.2), CD57 (HNK-1), TCRαβ (WT31) and TCRγδ (11F2). All antibody conjugates were from BD Biosciences (San Jose, CA, USA). A 4-color flow cytometric immune phenotype strategy, antibodies to CD3, CD8 and the IO Test Beta Mark TCRVβ Repertoire kit (Beckman Coulter, Marseille, France) were used. Analyses were done as described [11].

### Separation of mononuclear cells and DNA isolation

Blood or bone marrow samples were collected at diagnosis and mononuclear cells isolated by density gradient centrifugation using Ficoll-Hypaque. Genomic DNA was extracted and purified as described using the QIAamp blood kit (Qiagen, Hilden, Germany). DNA concentration and purity were measured with Eppendorf Biophotometer (Eppendorf, Hamburg, Germany).

### Multiplex polymerase chain reaction (PCR) analysis for T-cell receptor gene rearrangement

PCR analysis for T-cell receptor gene rearrangement was performed using genomic DNA extracted from the mononuclear cells and performed as previously described [12].

### Analysis of STAT3 mutations

DNA from samples from 26 consenting subjects was tested for mutations in exons 20 and 21. STAT3 amplicon sequencing and data analyses were done as described [11].

### Treatment

Indications for treatment included: (1) absolute neutrophil level (ANC) < 0.5 × 10E + 9/L; (2) recurrent infections independent of ANC; (3) symptomatic or RBC-transfusion-dependent anemia; and/or (4) autoimmune conditions such as PRCA or rheumatoid arthritis [13]. Methotrexate was given orally at a dose of 10 mg/mE + 2 weekly. Oral low-dose folic acid (5 mg/d) was given to prevent mouth ulcers. Prednisone (0.5–1 mg/kg/d) could be given for ≤ 2 months of starting methotrexate.

### Response criteria

Complete response was defined as normalization of blood cell levels and reduction of LGLs to the normal range (ANC > 1.5 × 10E + 9/L; lymphocytes < 4.0 × 10E + 9/L; hemoglobin concentration >110 g/L; platelets >100 × 10E + 9/L). Partial response was defined when either of the following: ANC increase > 50% and > 0.5 but < 1.5 × 10E + 9/L; haemoglobin concentration increase > 20 g/L and RBC-transfusion–independence but < 110 g/L. No response was defined as a response < partial response > 4 months after starting methotrexate but without disease progression. Progressive disease was defined as worsening of hematologic parameters in subjects previously achieving ≥ partial response. Toxicity was graded using the modified NCI Common Terminology Criteria (CTC, version 4.0). Blood cell levels and differentials and kidney and liver function tests were analyzed monthly. Treatment was continued in responders until relapse, death or withdrawal of consent.

### Endpoints

Time-to-response was defined as the interval from starting methotrexate to a ≥ 50% improvement in blood cell level(s). Response duration was defined as the interval from declaring response to relapse. Treatment-free-survival was defined as the interval from date of diagnosis to starting methotrexate. Survival was defined as the interval from diagnosis to death or last follow-up.

### Statistical analyses

Statistical analyses used the SPSS program for Windows (version 16.0). Comparisons of proportions and ranks of variables between groups were performed by chi-squared-test, Fisher exact test or Student *t*-test. Kaplan-Meier survival estimates were constructed and differences compared by log-rank test. An effect was considered statistically significant at *P* < 0.05. *P*-values were two sided.
